# Waste to resource: use of water treatment residual for increased maize productivity and micronutrient content

**DOI:** 10.1007/s10653-021-01100-z

**Published:** 2021-09-27

**Authors:** T. Gwandu, L. I. Blake, H. Nezomba, J. Rurinda, S. Chivasa, F. Mtambanengwe, K. L. Johnson

**Affiliations:** 1 Department of Engineering, Durham University, Durham, DH1 3LE UK; 2 Department of Biological Sciences, Durham University, Durham, DH1 3LE UK; 3 Soil Fertility Consortium for Southern Africa (SOFECSA) Research Group, Department of Soil Science and Environment, University of Zimbabwe, Mount Pleasant, P.O. Box MP167, Harare, Zimbabwe

**Keywords:** Aluminium-based water treatment residual, Organic nutrient resources, Mineral fertilizer, Soil chemical properties, Maize dry matter yield, Nutrient uptake

## Abstract

Soil degradation, which is linked to poor nutrient management, remains a major constraint to sustained crop production in smallholder urban agriculture (UA) in sub-Saharan Africa (SSA). While organic nutrient resources are often used in UA to complement mineral fertilizers in soil fertility management, they are usually scarce and of poor quality to provide optimum nutrients for crop uptake. Alternative soil nutrient management options are required. This study, therefore, evaluates the short-term benefits of applying an aluminium-based water treatment residual (Al-WTR), in combination with compost and inorganic P fertilizer, on soil chemical properties, and maize (*Zea mays* L.) productivity and nutrient uptake. An eight-week greenhouse experiment was established with 12 treatments consisting of soil, Al-WTR and compost (with or without P fertilizer). The co-amendment (10% Al-WTR + 10% compost) produced maize shoot biomass of 3.92 ± 0.16 g at 5 weeks after emergence, significantly (*p* < 0.05) out-yielding the unamended control which yielded 1.33 ± 0.17 g. The addition of P fertilizer to the co-amendment further increased maize shoot yield by about twofold (7.23 ± 0.07 g). The co-amendment (10% Al-WTR + 10% C) with P increased maize uptake of zinc (Zn), copper (Cu) and manganese (Mn), compared with 10% C + P. Overall, the results demonstrate that combining Al-WTR, compost and P fertilizer increases maize productivity and micronutrient uptake in comparison with single amendments of compost and fertilizer. The enhanced micronutrient uptake can potentially improve maize grain quality, and subsequently human nutrition for the urban population of SSA, partly addressing the UN’s Sustainable Development Goal number 3 of improving diets.

## Introduction

There is growing concern over food and nutrition insecurity in the urban communities of Southern Africa, due to rapid human population growth coupled with limited job opportunities against limited livelihood alternatives (Awad, 2019; Cockx et al., 2018). To cope with these changes, many urban dwellers in the region are increasingly resorting to urban agriculture (UA) for household food, nutrition and income security (Kutiwa et al., 2010; Takavarasha, 2003). However, as is the case in many rural communities in Southern Africa (Kamanga et al., 2014; Mapfumo & Giller, 2001), crop production has remained low in urban areas due to a combination of factors, including declining soil fertility (Mtangadura et al., 2017; Nyamasoka et al., 2015) and a changing climate (Rurinda et al., 2015), hampering efforts towards achievement of Sustainable Development Goals, most of which are underpinned by soil health (Keesstra et al., 2016; Lal, 2019). Without addressing poor soil fertility and the negative impacts of the changing climate, crop yields will remain poor, increasing the number of households vulnerable to food deficits.

Although mineral fertilizer is important for rebuilding soil nutrient stocks and increased crop productivity (Kihara et al., 2020; Rurinda et al., 2020), many farmers in SSA have limited or no access to mineral fertilizer due to high costs and inaccessibility. Current fertilizer application rates in SSA average only about 16 kg ha^−1^. year^−1^, compared with over 100 kg ha^−1^. year^−1^ in Europe and North America and over 150 kg ha^−1^. year^−1^ in China (FAOSTAT, 2019). To increase and maintain crop production in SSA, use of locally available organic nutrient resources is important (Mapfumo & Giller, 2001). Organic nutrient resources increase crop yields by supplying plant nutrients in the short to medium term while improving soil organic matter and other soil physicochemical and biological properties in the long term (Mtambanengwe & Mapfumo, 2005). Farmers in rural areas of SSA rely on locally available nutrient resources such as partially composted woodland litter and livestock manure for crop production (Manzeke et al., 2012; Mapfumo & Giller, 2001). In urban communities, crop residues from previous harvests are the most available organic nutrient resource because of little competition for their use as livestock feed. However, some farmers prefer to burn the crop residues due to the drudgery involved during their incorporation. Water treatment residual (WTR) is a potential organo-mineral resource that could be used for soil fertility improvement and soil health in UA, but its potential use remains largely untapped. WTR is a by-product of the municipal clean water treatment process, which is organo-mineral, containing aluminium (Al) and/or iron (Fe) oxides, activated carbon and flocculated material from reservoirs, including clay particles, mineral nutrients and organic matter (Elliot et al. 1990; Matilainen et al., 2010). WTRs can potentially contribute to soil carbon build-up in the long term because the organic matter becomes tightly bound in the Fe and Al oxide matrix (Elliott & Dempsey, 1991; Novak & Watts, 2004). When WTR is added to soil, the resultant soil organic matter (SOM) is adsorbed into the mineral matrix and is thus protected from microbial attack (Kögel‐Knabner et al. 2008). On a global scale, it is estimated that 10,000 t of WTR, on average, are produced daily from standard water treatment works (Ahmad et al., 2016; Gibbons & Gagnon, 2011). While information on WTR production trends from Africa is largely missing, given the rapid urbanization, more water will be purified to meet the increasing human demand, and inevitably more WTR will be generated. Since the WTR contains mineral nutrients and organic matter, it can, therefore, be used as an alternative source of soil nutrients including micronutrients for plant nutrition and soil health in UA. Use of WTR as a soil amendment can minimize costs of its disposal and the undesirable impacts on the environment.

Research has been done to understand the potential of WTR as a soil ameliorant (Dassanayake et al., 2015; Ippolito, 2015). Of major concern, however, is phosphorus (P) dynamics following the addition of WTR to soil. Phosphorus is an important macronutrient in plant growth (Malhotra et al., 2018) and is one of the most limiting nutrients in the predominantly sandy soils of Southern Africa (Rurinda et al., 2020). Jonasson (1996) and Cox et al. (1997) demonstrated that Al or Fe oxides present in WTR potentially bind P in soil, making it unavailable for plant uptake. On the contrary, studies by Grabarek and Krug (1987), and Geertsema et al. (1994) have shown that the application of WTR to soil has no effect on P uptake and plant growth in tree species. Other reports (Mahdy et al., 2007; Rengasamy et al., 1980) have confirmed improved soil properties and dry matter yields of maize in fertilized and unfertilized pots amended with WTRs, albeit at certain threshold application levels. However, this also differed with soil type (Mahdy et al., 2007). Evaluating options that reduce the P-fixing ability of WTR would be key for sustainable use of WTR in crop production. Co-application of WTR with P fertilizer may eliminate the problem of P deficiencies for plant growth (Hyde & Morris, 2004). Alternatively, co-application of WTR with compost or other organic plant or animal-based waste may help to alleviate P sorption by the Fe and Al oxides in soils (Havlin et al., 2005). Hsu and Hseu (2011) reported an increase in shoot biomass production of Bahia grass (*Paspalum notatum*) without changes in soil P availability due to co-application of WTR and pine bark compost. Recent work in Southern Africa has also proven that when WTR is used in combination with organic compost with a 1:1 co-application ratio, wheat (*Triticum aestivum*) productivity increased by 33% (Clarke et al., 2019). The resultant wheat growth was attributed to balanced nutrition, with P and potassium (K) from the compost and nitrogen (N) from WTR. However, this has not yet been tested in maize (*Zea mays* L.), a strategic crop for food security in Southern Africa, including Zimbabwe. The overall hypothesis was that the application of WTR in combination with compost and P fertilizer improved soil chemical properties, maize nutrient uptake and dry matter yield relative to unfertilized maize. The objective of this study was to understand the effects of co-applying Al-WTR, compost and inorganic P fertilizer, on soil chemical properties, and maize (*Zea mays* L.) productivity and nutrient uptake.

## Materials and methods

### Experimental set-up

An eight-week greenhouse pot experiment was set up at Durham University (54° 46′ 22.80″ *N*, − 1° 34′ 26.40″ *W*), UK. The experiment consisted of 12 treatments as shown in Table 1.

**Table 1:** Experimental treatments

Treatment number	Treatment composition
1	Control (Unamended soil)
2	10% Al-WTR
3	10% compost
4	20% Al-WTR
5	20% compost
6	10% Al-WTR + 10% compost
7	Standard NPK (soil amended with NPK)
8	10% Al-WTR + P
9	10% compost + P
10	20% Al-WTR + P
11	20% compost + P
12	10% Al-WTR + 10% compost + P

A sandy-loam soil from Zimbabwe was used in the experiment. The soil is broadly classified as a Lixisol (WRB, 2006), exhibits low inherent fertility especially nitrogen (N), phosphorus (P), carbon (C) and sulphur (S) and is characterized by low water holding capacity (Nyamapfene, 1991). Lixisols are prone to run-off and known to readily compact and crust under natural rainfall and are thus drought sensitive. This soil typifies most soils found in smallholder farming systems of Zimbabwe and most parts of Southern and West Africa (Nyamapfene, 1991; FAO.I. ISSS, 1998). A peat-based commercial compost used in this study was sourced locally in the UK. The Al-WTR was sourced from Carmoney Water Treatment Works, Northern Ireland. Al-WTR is also commonly available in Zimbabwe, where most water treatment works use aluminium sulphate (alum) in their water treatment processes. The physical and chemical characteristics of Al-WTR from Prince Edward waterworks (Zimbabwe) were comparable to the Carmoney Al-WTR. All the three materials (soil, compost and Al-WTR) were sieved to 2 mm for characterization of their physical and chemical properties and used in the pot trial.

The soil was limed to a target pH of 5.5, which is favourable for maize growth. The different soil mixtures were incubated for three weeks during which they were watered to field capacity. After three weeks, they were then transferred into one litre PVC-plastic pots with perforated bases to allow free drainage of excess water. The pots were arranged in a completely randomized design (CRD) with 6 replicates per treatment. One seed of maize variety SC513 (137 days to maturity), commonly grown in Zimbabwe, was planted in each pot. The greenhouse temperature was maintained at 24 °C, and lighting was supplemented with artificial light set on a 16-h photoperiod for the duration of the experiment until harvest. Throughout the growth period, watering was done to maintain the soils’ field capacity. For treatments with P, a compound fertilizer, Compound D (7% N, 14% P_2_O_5_, and 7% K_2_O) from Zimbabwe was used as a source of available P applied by spreading on soil and mixing-in to a depth of 5 cm before planting. Fertilizer rates were differentially applied across treatments based on the targeted P rates of 26 kg P ha^−1^ (2.67 g.pot^−1^) for treatment 7 (standard NPK) and a target of 14 kg P ha^−1^ (1.44 g.pot^−1^) for compost and WTR treatments, following P fertilization rates recommended by Mtambanengwe and Mapfumo (2009). Except for the unamended control (treatment 1), all treatments received additional N in the form of ammonium nitrate (34.5% N), as topdressing at a rate of 90 kg N ha^−1^, and this was applied at 3 weeks after emergence.

### Analysis of materials used in the experiment

The pH of the material was measured with 0.01 M CaCl_2_ (Anderson & Ingram, 1993) and readings taken using a standard pH meter (Hanna, H18424). Electrical conductivity (EC) was determined using the water extraction method and readings taken using the conductivity meter (Jenway470JCO2). Exchangeable bases (Ca, Mg and K) were extracted using 1 M ammonium acetate (Anderson & Ingram, 1993), whilst available P was extracted using 0.5 M NaHCO_3_ and all were measured using an inductively coupled plasma optical omission spectrometry (Agilent 5100 ICP-OES). Exchangeable acidity was determined through titration using phenolphthalein indicator. Total C and N were determined by combustion using flash 2000 organic elemental analyser. The metals, manganese (Mn), sodium (Na), zinc (Zn), copper (Cu), aluminium (Al), iron (Fe), magnesium (Mg), calcium (Ca) and potassium (K), were determined by X-ray fluorescence (XRF) via fused bead and wax pellet (Fitton, 1997).

### Maize growth measurements, nutrient uptake and residual soil chemical analysis

Weekly measurements of plant height and number of leaves were conducted for five (5) consecutive weeks beginning on the 7th day after emergence. Plant height was measured using a tape measure from the soil surface to the highest point of the arch of the uppermost leaf with its tip pointing down. The number of leaves was determined by physical counting based on the leaf tip method (Manitoba Crop Reports, 2020). The leaf tip method involves counting all leaves, including any leaf tips that have emerged from the whorl at the top of the plant. On the 35th day, maize plants were cut just above the soil surface to separate shoots and roots. Both the shoots and roots were washed in distilled water and left for 4 days under shade for air drying. After the 4 days, the biomass was oven-dried at 65 °C until a constant weight was reached. Total dry shoot and root biomass were then determined. The above-ground biomass (shoots) were ground to pass through a 2-mm sieve using a magic bullet nutri-blender (EAN: 5,060,191,467,360) for determination of total N, P, K, Ca, Mg Cu, Mn, Zn, Al, Pb and Ni. Total N was analysed using the Thermo Scientific Flash 2000 Organic Elemental Analyser, whilst P was extracted using the bicarbonate method (Olsen, 1954) and analysed using an ICP-OES (Agilent 5100). Ca, Mg, K, Cu, Mn, Zn, Al, Pb and Ni were extracted using the microwave-assisted aqua-regia digestion method (Eskilsson & Björklund, 2000) and concentrations read using the ICP-OES. Nutrient uptake is calculated with Eq. (1)

1 $$\begin{aligned} {\text{Nutrient }}X{\text{ }}\left( {{\text{mg}}/{\text{kg}}} \right){\text{ }} = & {\text{ }}\left[ \begin{gathered} \left( {X{\text{ concentration }}\left( {{\text{mg l}}^{{ - 1}} } \right)/1000} \right){\text{ }} \hfill \\ \times {\text{ volume of the sample used }}\left( {{\text{ml}}} \right) \hfill \\ \end{gathered} \right]{\text{ }} \\ / & {\text{ }}\left[ {{\text{sample weight }}\left( {\text{g}} \right){\text{ }}/1000} \right] \\ \end{aligned}$$

where *X* is N, P, K, Ca, Mg, Zn, Cu, Ni, Mn, Pb or Al.

For N, P, Ca, Mg and K uptake was quantified in g kg^−1^, while for Zn, Cu, Pb, Ni, Al and Mn uptake was measured in mg/kg.

Chemical characteristics of the post-harvest soils were analysed as described in Sect. 2.2.

### Data analysis

Analysis of variance (ANOVA) for a completely randomized design was used to analyse the effects of amendments on maize plant growth, nutrient uptake and post-harvest soil chemical properties using GENSTAT 19th Edition. Duncan’s multiple-range test was then used to compare treatment means for all the measured parameters at *p* < 0.05.

## Results

### Chemical characteristics of soil, Al-WTR and compost

The soil used in this study had high sand content (73%), very low pH (4.0) and a relatively high exchangeable acidity (Table 2). The soil had low organic C and nutrient content, including total N, P, compared with both Al-WTR and compost. However, available P in the soil (6 mg kg^−1^) was slightly higher than in the Al-WTR (5 mg kg^−1^) (Table 2). The low levels of cations in the soil were also consistent with a low CEC. The compost used in the study had a high nutrient content in general and a very high CEC, but low pH and a high C:N (Table 2). The Al-WTR, on the other hand, had a moderate pH (pH 5.7), which is favourable for maize production. The Al-WTR also had total N, which was equivalent to compost averaging 1.28%.

**Table 2:** Chemical characteristics of soil, compost and WTR used in the experiment

Parameter	*Soil	*Al-WTR^a^	*Compost	European Community maximum limit^2^
Sand (%)	73	ND	ND	
Silt (%)	5	ND	ND	
Clay (%)	22	ND	ND	
pH (0.01 m CaCl_2_)	4.0	5.7	4.8	
EC (µS cm^−1^)	80	872	2010	
Exchangeable acidity (meq/100 g)	6.0	2.5	10.5	
CEC(cmol( +)kg^−1^	6.5	31	84.3	
Total P (%)	0.06	0.12	0.10	
Available P (mgkg^−1^)	6	5	261	
Total N (%)	0.03	1.28	1.28	
Total organic C (%)	0.47	18.37	46.9	
C/N ratio	15.7	14	36.7	
Ca (meq/100 g)	0.5	2.9	55.9	
Mg (meq/100 g)	0.3	0.2	12.5	
K (meq/100 g)	0.1	0.1	5.4	
Pb (mg kg^−1^)	4.1	17.6	7.5	750
Cu (mg kg^−1^)	0.4	45.7	5.7	200
Zn (mg kg^−1^)	0.5	203.8	35.4	400
Ni (mg kg^−1^)	5.1	41.0	2.8	150
Mn (mg kg^−1^)	29	4534	156	ND
Al (g kg^−1^)	1.2	15.2	2.2	ND

ND-not determined

^a^*Al-WTR* aluminium water treatment residual; *EC* electrical conductivity; *CEC* cation exchange capacity

### Effects of different treatments on maize growth and biomass partitioning

**Fig. 1:**
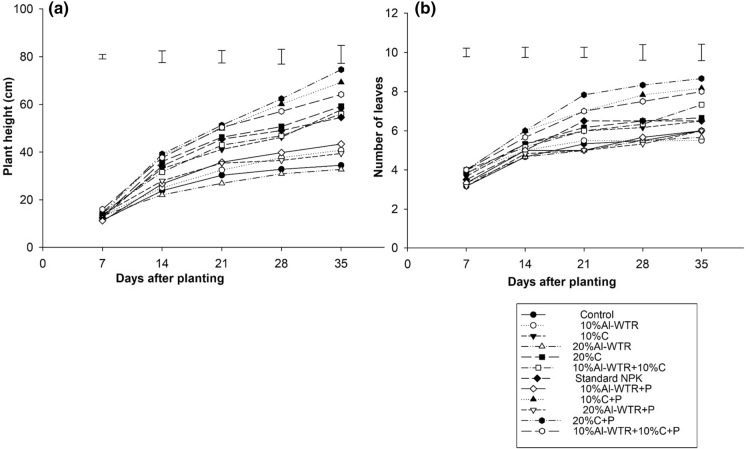
Effects of different soil amendments on maize plant height (**a**) and mean number of leaves (**b**), C-compost; C + P-compost + inorganic basal P; Al-WTR-aluminium water treatment residual; Al-WTR + P—aluminium water treatment residual + inorganic basal P; Std NPK-standard inorganic fertilizer consisting of compound D and ammonium nitrate. Error bars denote standard errors of the differences between means (SED) (*n* = *6)*

A slow growth response of plant height to all treatments was observed until day 14; thereafter, a sudden increase in plant height was observed for compost treatments, the co-amendment and standard NPK (Fig. 1a). At 35 days after planting, the maize plant height was 60.17 ± 1.2 cm for the co-amendment, 10% Al-WTR + 10% C + P, which was significantly higher than 40.83 ± 3.5 cm and 54.58 ± 1.6 cm observed for the unamended control and standard NPK, respectively. Maize plant height for 10% Al-WTR + 10% C + P, 10% C + P (69 ± 1.8 cm) and 20% C + P (70 ± 1.8 cm) was comparable (Fig. 1a). Number of leaves also followed a similar trend to plant height in both instances (Fig. 1b). Both the plant height and leaf number decreased with an increased concentration of Al-WTR from 10 to 20% (Fig. 1). Except in Al-WTR treatments, the addition of P fertilizer resulted in significant increase in plant height for all treatments. The addition of P fertilizer had no influence in number of leaves except that they were only smaller in size in treatments without P (Fig. 1b).

**Fig. 2:**
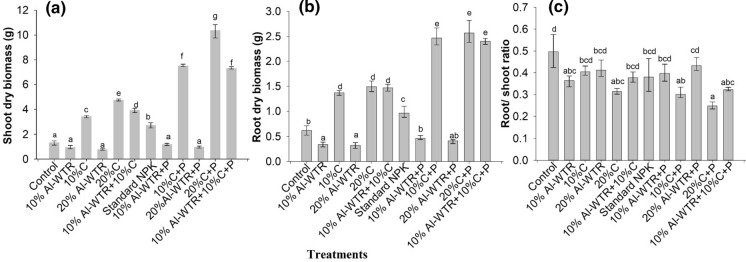
Shoot (**a**) and root (**b**) dry matter accumulation and root/shoot ratios (**c**) for different soil amendments at 5 weeks after emergence. Bars represent mean ± SE (*n* = 6). Bars with different letters are significantly different at *p* < 0.05

Maize above-ground (shoot) dry matter accumulation was highest (10.67 ± 0.55 g) in the 20% C + P treatment, whilst the least (0.76 ± 0.07 g) was observed for the 20% WTR (Fig. 2a). The co-amendment, 10% Al-WTR + 10% C significantly (p < 0.05) yielded 3.92 ± 0.16 g higher shoot biomass than the unamended control which produced 1.33 ± 0.17 g. The addition of P fertilizer to the co-amendment (10% Al-WTR + 10% C) further increased maize dry matter yield about twofold (7.23 ± 0.07 g) (Fig. 2a). There was, however, no significant difference in maize shoot dry matter biomass between 10% Al-WTR + 10% C + P (7.23 ± 0.07 g) and 10% C + P, which yielded 7.5 g ± 0.10 g (Fig. 2a). The co-amendment of 10% Al-WTR + 10% C also yielded significantly (*p* < 0.05) higher shoot biomass compared with 10% C and standard NPK. Except for sole Al-WTR treatments, the addition of P fertilizer significantly (p < 0.05) increased shoot biomass yield across all treatments (Fig. 2a).

The highest root dry matter accumulation was attained in the treatment 20% C + P with 2.57 ± 0.22 g, but this did not differ significantly with 10% WTR + 10% C + P with 2.4 ± 0.07 g and 10% C + P with 2.45 ± 0.17 g (Fig. 2b). Likewise, root dry matter in the 10% C, 20% C and 10% Al-WTR + 10% C treatments did not differ statistically. Contrary to shoot biomass, the control yielded higher root biomass at 0.62 ± 0.09 g compared with 10% and 20% Al-WTR treatments both yielded < 0.35 g (Fig. 2b). Consistent with shoot biomass, both 10% Al-WTR + 10% C + P and 10% Al-WTR + 10% C yielded significantly (*p* < 0.05) higher root biomass relative to standard NPK (Fig. 2b). The addition of P fertilizer significantly increased root biomass yield across all treatments.

The control had the highest root-to-shoot ratio with 0.5 ± 0.02, whilst 20% C + P had the least at 0.25 ± 0.01 with the rest coming in between (Fig. 2c). Root-to-shoot ratios were generally low in both 10% Al-WTR + 10% C + P and 10% Al-WTR + 10% C compared with sole Al-WTR and the control (Fig. 2c). However, similar root/shoot ratios were observed in 10% Al-WTR + 10% C + P, and 10% C + P (Fig. 2c). Overall, these data revealed that the co-amendment resulted in higher maize growth (plant height, number of leaves and dry matter accumulation) relative to the unamended control, standard NPK and sole Al-WTR treatments.

### Uptake of nitrogen (N) and phosphorus (P) by maize

**Fig. 3:**
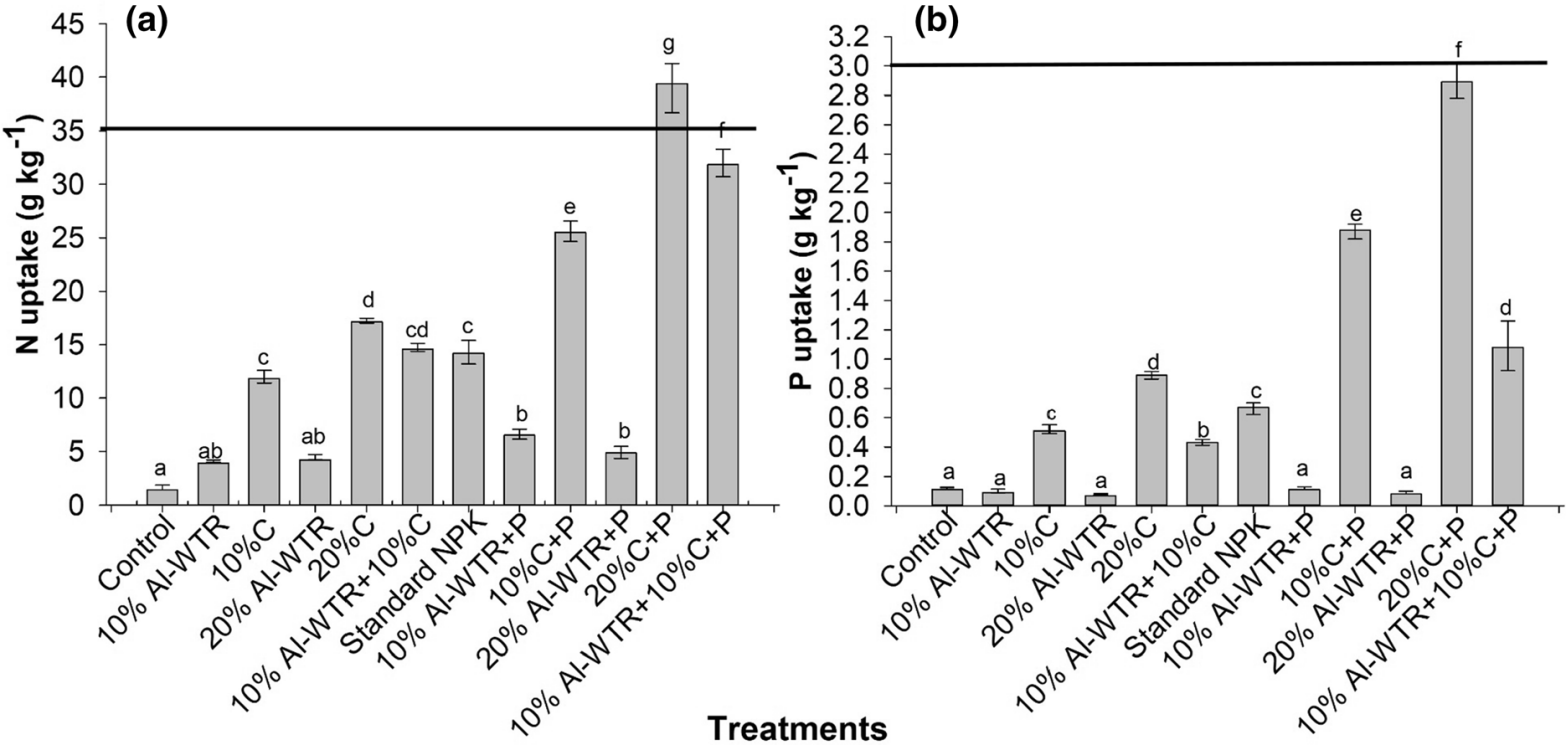
Total N (**a**) and P (**b**) uptake by maize for different soil amendments at 5 weeks after emergence. The solid horizontal lines represent the critical N and P levels in maize tissue (Tandon, 1993). Bars are mean ± se (*n* = 3). Means with the same letter do not differ significantly at *p* < 0.05

Except for 20% C + P with 39.38 ± 0.01 g N kg^−1^, N uptake for the co-amendment 10% Al-WTR + 10% C + P of 31.86 ± 0.01 g N kg^−1^ was significantly (*p* < 0.05) higher than for the rest of the treatments (Fig. 3a). The least N uptake was observed in the unamended control with 1.43 ± 0.01 g N kg^−1^ (Fig. 3a). Nitrogen uptake in the control, however, did not differ for both 10 and 20% Al-WTR treatments. Addition of P fertilizer had a significant influence on N uptake by maize across all treatments except for the sole Al-WTR treatments. Only the treatment 20% C + P exceeded the critical N limit in maize plant tissue (Fig. 3a).

There was a contrasting trend in P uptake relative to N uptake. Uptake of P for both co-amendments of 10% Al-WTR + 10% C + P (1.08 ± 0.08 g P kg^−1^) and 10% Al-WTR + 10% C (0.43 ± 0.06 g P kg^−1^) was significantly (*p* < 0.05) higher than for the unamended control with 0.11 ± 0.04 g P kg^−1^ (Fig. 3b). However, both 10 and 20% compost treatments (± P) resulted in significantly higher P uptake compared with 10% Al-WTR + 10% C and 10% Al-WTR + 10% C + P (Fig. 3b). Consistent with N uptake, 10% Al-WTR + 10% C + P had significantly higher P uptake compared with standard NPK, which attained 0.67 ± 0.07 g P kg^−1^. Although not significantly different, the uptake of P declined with increase from 10 to 20% Al-WTR levels. The addition of P fertilizer did not result in significant changes in P uptake in Al-WTR treatments (Fig. 3b). Phosphorus uptake across all treatments fell below the critical limit for P (3 g kg^−1^) (Fig. 3b).

Generally, results revealed that the addition of P fertilizer resulted in improved uptake of N and P by maize across all treatments except for sole Al-WTR treatments. P uptake was lower across all treatments in comparison with N (Fig. 3).

### Effects of different soil amendments on soil chemical properties at harvest

Post-harvest soil pH due to sole Al-WTR treatments and both the 10%Al-WTR + 10%C and 10%Al-WTR + 10%C + P was comparable, whilst all compost treatments had a significantly lower pH (Table 3). This is possibly because the compost used in the experiment had very low pH (see Table 2). Electrical conductivity (EC) in 10%Al-WTR + 10%C + P (1.79 ±0.07) was comparable to 20%C (1.84 ±0.07) and significantly (p <0.05) higher relative to the unamended control, sole WTR and standard NPK (Table 3). Although compost treatments had a significantly higher CEC compared to the rest of the treatments, both 10% Al-WTR + 10% C and 10% Al-WTR + 10% C + P, in turn had significantly higher CEC in comparison with the unamended control, standard NPK and sole WTR treatments. 10% Al-WTR +10% C + P had the highest P content (0.083% ± 1.1) whilst the control (0.04 % ± 0.03) had the lowermost (Table 3).

**Table 3:** Effects of different soil amendments on soil chemical properties at harvest

Parameter	Control	10%WTR	10%C	20%WTR	20%C	10%WTR + 10%C	Std NPK	10%WTR + P	10%C + P	20%WTR + P	20%C + P	10%WTR + 10%C + P
pH	6.4 ± 0.06 cd	6.8 ± 0.18d	5.6 ± 0.03b	6.4 ± 0.06 cd	5.0 ± 0.03a	6.3 ± 0.5 cd	6.8 ± 0.03d	6.8 ± 0.003d	5.2 ± 0.03ab	6.2 ± 0.1c	4.9 ± 0.08a	6.3 ± 0.03 cd
EC (dSm^−1^)	0.27 ± 0.07a	0.59 ± 0.07b	1.18 ± 0.17cde	0.77 ± 0.07b	1.59 ± 0.07 fg	1.46 ± 0.07ef	1.42 ± 0.07def	1.07 ± 0.07c	1.33 ± 0.07cdef	1.15 ± 0.07 cd	1.84 ± 0.07 g	1.79 ± 0.07 g
CEC (cmol_(+)_kg^−1^)	4.33 ± 0.33a	5 ± 0.00a	14.33 ± 0.88b	6.67 ± 0.88a	23.67 ± 0.88ef	16.33 ± 0.33bc	4 ± 0.00a	5 ± 0.00a	21.67 ± 0.33de	7 ± 0.57a	26 ± 0.33f	18.5 ± 0.33 cd
Total P (%)	0.042 ± 0.3a	0.049 ± 0.7d	0.045 ± 0.6b	0.055 ± 0.7e	0.048 ± 0.3c	0.057 ± 1.5 g	0.068 ± 0.7j	0.062 ± 0.7 h	0.057 ± 0.6f	0.074 ± 0.6 k	0.066 ± 0.9i	0.083 ± 1.1 l
Total N (%) Total C (%)	0.03 ± 0.00a 0.41 ± 0.01 h	0.17 ± 0.05b 2.09 ± 0.14 g	0.19 ± 0.05b 3.83 ± 0.28e	0.25 ± 0.05bcd 3.69 ± 0.20ef	0.29 ± 0.02cde 7.82 ± 0.38a	0.35 ± 0.04e 7.64 ± 0.05a	0.07 ± 0.003a 0.47 ± 0.27 h	0.21 ± 0.02bc 2.23 ± 0.38 g	0.17 ± 0.01b 4.73 ± 0.4d	0.30 ± 0.03de 3.42 ± 0.39f	0.26 ± 0.01bcde 7.22 ± 0.5b	0.32 ± 0.02de 6.47 ± 0.3c
Ca (g kg^−1^)	5.69 ± 1.6ab	6.0 ± 1.5abc	5.9 ± 0.5abc	5.5 ± 0.6ab	8.3 ± 0.6bcd	7.8 ± 0.7abcd	9.9 ± 1.5d	6.2 ± 0.5abc	7.4 ± 0.4abcd	5.06 ± 0.4a	8.7 ± 0.7 cd	6.9 ± 0.23abc
Mg (g kg^−1^)	0.4 ± 0.01a	0.5 ± 0.004a	1.6 ± 0.18b	2.0 ± 0.35bc	2.3 ± 0.11c	1.6 ± 0.24b	0.4 ± 0.02a	0.5 ± 0.005a	2.0 ± 0.1bc	0.6 ± 0.008a	2.5 ± 0.1c	2.2 ± 0.1c
K (g kg^−1^)	10.7 ± 0.32 cd	10.6 ± 0.23 cd	9.9 ± 0.01abc	9.2 ± 0.21ab	8.9 ± 0.13a	9.4 ± 0.32ab	11.4 ± 0.43d	10.7 ± 0.33 cd	9.4 ± 0.31ab	9.5 ± 0.14ab	8.8 ± 0.11a	10.0 ± 0.30bc
Zn (mg kg^−1^)	15.3 ± 0.29a	41.0 ± 0.32f	18.1 ± 0.15b	58.1 ± 0.1 h	21.3 ± 0.3c	46.1 ± 0.15 g	21.1 ± 0.0c	41.0 ± 0.06f	23.4 ± 0.32d	62.2 ± 0.15i	25.5 ± 0.23e	46.1 ± 0.13 g
Pb (mg kg^−1^)	18.5 ± 0.89abc	20.3 ± 0.46 cd	17.1 ± 0.61ab	19.3 ± 0.34bcd	16.6 ± 0.46a	17.2 ± 1.00ab	20.3 ± 0.65 cd	18.9 ± 0.6bcd	17.7 ± 1.07ab	20.1 ± 0.03 cd	18.5 ± 0.89abc	20.9 ± 0.55d
Al (g kg^−1^)	3.57 ± 0.12d	4.64 ± 0.35f	3.55 ± 0.33d	3.080 ± 0.03b	2.92 ± 0.30a	5.24 ± 0.03i	4.01 ± 0.32e	4.82 ± 0.03 g	3.09 ± 0.09b	5.45 ± 0.33j	3.15 ± 0.32c	4.91 ± 0.35 h
Cu (mg kg^−1^)	4.9 ± 0.28a	11.0 ± 0.12e	6.1 ± 0.03b	18.4 ± 0.33 g	9.5 ± 0.2c	16.2 ± 0.15f	6.5 ± 0.11b	10.57 ± 0.13e	10.6 ± 0.12e	10.1 ± 0.07d	9.6 ± 0.15 cd	15.7 ± 0.09f
Ni (mg kg^−1^)	9.13 ± 0.78a	14 ± 0.11b	13.8 ± 0.7b	16.2 ± 0.87bcd	15.3 ± 3.6bc	17.9 ± 2.2 cd	10.7 ± 0.12a	14.5 ± 0.47b	14.03 ± 0.84b	17.6 ± 0.03 cd	14.6 ± 0.26b	18.9 ± 0.54d
Mn (mg kg^−1^)	338.3 ± 0.33a	1042 ± 0.58 h	408 ± 0.03c	1338.3 ± 0.33 k	410.4 ± 0.32d	1233.7 ± 0.33i	471 ± 0.03f	1287.3 ± 0.33j	404.3 ± 0.33b	1353.3 ± 0.32i	423.1 ± 0.03e	1002 ± 0.03 g

Data are means ± standard error of the means for the three replicates. Mean data followed by different letters within the same row are significantly different at 5% level according to Duncan’s multiple-range test

Even though, residual soil basic cations (Ca and Mg) were generally higher in compost treatments, both 10% Al- WTR + 10% C and 10% Al-WTR + 10%C + P had significantly (p < 0.05) higher Ca and Mg than the control (Table 3). Contrastingly, soil residual K was significantly higher in soil only treatments - the control and standard NPK as compared to the rest of the other treatments. There were also significantly (P < 0.05) higher levels of residual Zn, Cu and Mn in sole Al-WTR treatments compared to the rest of the other treatments (Table 3). Residual Pb and Ni were comparable among 10%Al-WTR + 10%C + P, Al-WTR treatments and standard NPK. 20%Al-WTR + P had significantly (P < 0.05) higher Al levels as compared to the rest of the treatments. However, the post-harvest metal levels were lower than the maximum limits for the metals in agricultural soils (see table 1).

### Uptake of basic cations by maize

**Fig. 4:**
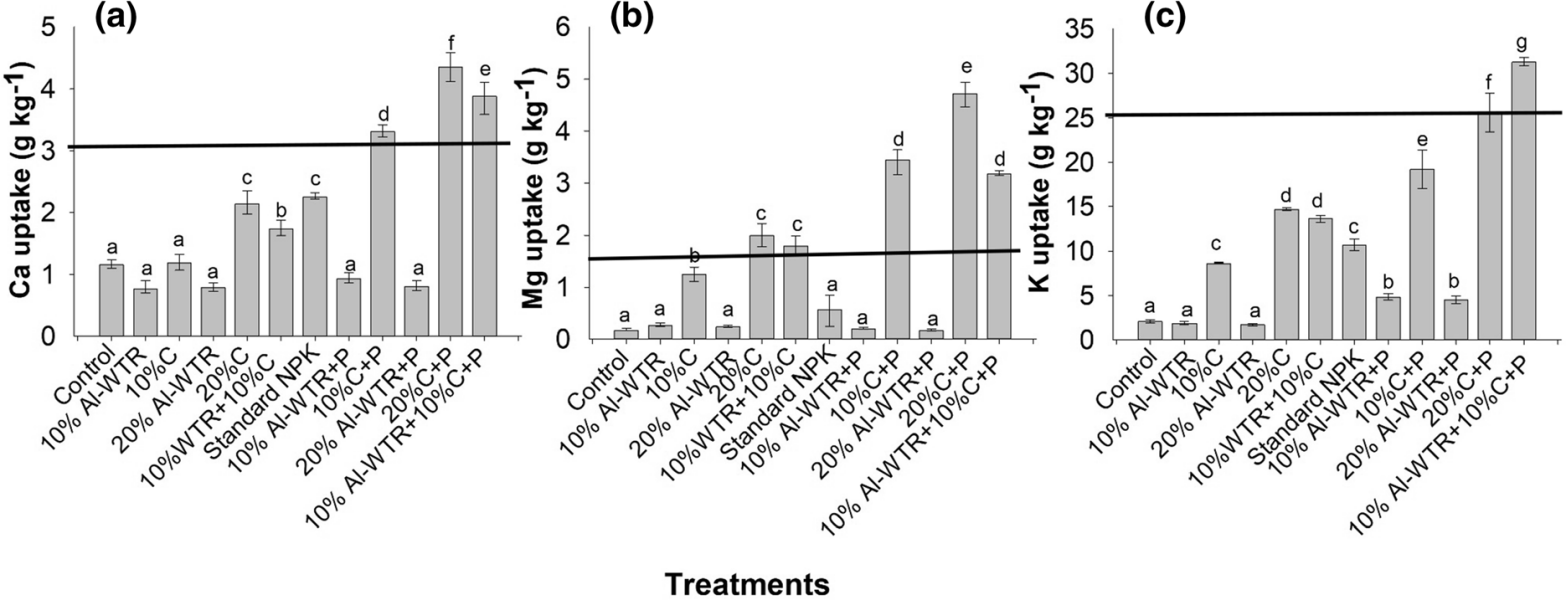
Mean values of Ca (**a**), Mg (**b**) and K (**c**) uptake by maize at 35 days after emergence. The solid horizontal lines represent critical limits for Ca, Mg and K in maize plant tissue (Tandon 1993). Bars are mean ± SE (*n* = 3). Means that do not differ significantly at *p* < 0.05 contain the same letter

Except for the treatment 20% C + P (4.35 ± 0.17 g Ca kg^−1^), the co-amendment of 10% Al-WTR + 10% C + P (3.88 ± 0.23 g Ca kg^−1^) resulted in higher Ca uptake by maize compared with the rest of the treatments (Fig. 4a). The lowest uptake was in 20% Al-WTR with 0.79 ± 0.58 g Ca kg^−1^. The addition of P fertilizer resulted in an increase in the uptake of Ca across all treatments except sole Al-WTR treatments (Fig. 4a). The co-amendment, 10% Al-WTR + 10% C + P; 10% C + P and 20% C + P attained more than 3 g Ca kg^−1^; a value which is above the critical Ca level required in maize plant tissue.

Uptake of Mg followed a similar trend to Ca, with 20% C + P consistently attaining the highest uptake. The co-amendment, 10% Al-WTR + 10% C + P in turn attained a higher Mg uptake than the control (0.17 ± 0.01 g Mg kg^−1^) and standard NPK (Fig. 4b). Similarities in the uptake of Mg were observed for 10% Al-WTR + 10% C + P and 10% C + P; 10% Al-WTR + 10% C and 10% C and for standard NPK, the control and Al-WTR treatments (Fig. 4b). Except for the Al-WTR treatments, the addition of P fertilizer increased the uptake of Mg across all treatments. Overall, the co-amendment (10% Al-WTR + 10% C and 10% Al-WTR + 10% C + P), and the compost treatments (± P) exceeded 1.5 g Mg kg^−1^, the critical Mg level in maize plant tissue.

Contrasting to Ca and Mg uptake, the highest K uptake was observed for the co-amendment, 10% Al-WTR + 10% C + P, which attained 31.25 ± 0.29 g K kg^−1^, while the lowest was recorded for 20% Al-WTR with 1.72 ± 0.21 g K kg^−1^ (Fig. 4c). Both co-amendments, 10% Al-WTR + 10% C and 10% Al-WTR + 10% C + P, resulted in significantly (*p* < 0.05) higher K uptake relative to the control and standard NPK. Uptake of K was comparable for 10% C and 10% Al-WTR + 10% C. Addition of P fertilizer had a positive influence in K uptake across all the treatments. The co-amendment, 10% Al-WTR + 10% C + P, was the only treatment that exceeded 25 g K kg^−1^, the critical limit of K in maize plant tissue. Uptake of K by maize was generally higher than Ca and Mg uptake (Fig. 4).

### Micronutrients uptake by maize

**Fig. 5:**
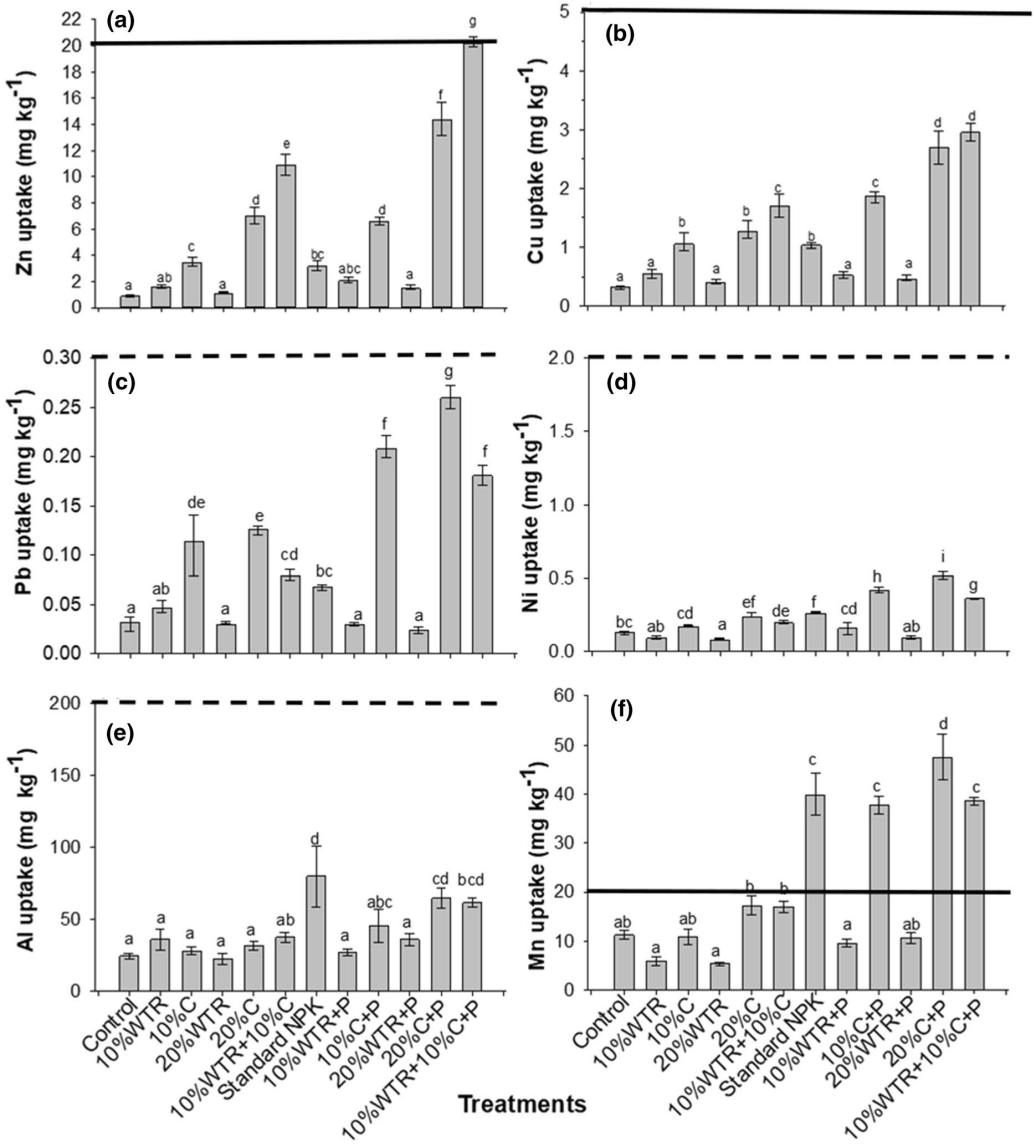
Average values of Zn (**a**) and Cu (**b**), Pb (**c**) and Ni (**d**), and Al (**e**) and Mn (**f**) uptake by maize at 5 weeks after emergence. The solid horizontal lines represent critical limits for Zn, Cu and Mn (Tandon 1993), while the broken lines represent toxicity thresholds for Pb (FAO/WHO 2001), Ni (WHO 1996) and Al (Pais and Jones Jr 1997). Bars are mean ± SE (*n* = 3). Means that do not differ significantly at *p* < 0.05 contain the same letter

The highest Zn uptake by maize, 20.19 ± 0.02 mg Zn kg^−1^, was observed for the co-amendment, 10% Al-WTR + 10% C + P, whilst the lowest was observed for the unamended control with 0.86 ± 0.1 mg Zn kg^−1^ (Fig. 5a). High Zn uptake by maize was also observed for the co-amendment (Fig. 5a). Uptake of Cu followed a similar trend to Zn, with the highest amounts observed for 10% Al-WTR + 10% C + P (2.95 ± 0.15 mg Cu kg^−1^). The control had the lowest uptake of 0.32 ± 0.03 mg Cu kg^− 1^ (Fig. 5b). Except for the sole Al-WTR treatments, the addition of P fertilizer generally increased Zn and Cu uptake across the treatments.

Pb uptake was largest in compost treatments with the highest value of 0.26 ± 0.01 mg Pb kg^−1^ observed for 20% C + P. There were no observed differences in Pb uptake between 10% Al-WTR + 10% C and standard NPK (Fig. 5c). Compared with compost treatments, 10% Al-WTR + 10% C + P resulted in reduced uptake of Pb (Fig. 5c). Consistent with Pb uptake, uptake of Ni followed a similar trend with 20% C + P yielding the highest uptake of 0.52 ± 0.02 mg Ni kg^−1^, whilst 20% Al-WTR had the least with 0.09 ± 0.01 mg Ni kg^−1^ (Fig. 5d) The co-amendment, 10% Al-WTR + 10% C + P, resulted in lower uptake of Ni by maize compared with 10% C + P. Except for Al-WTR treatments, the addition of P fertilizer resulted in an increase in Pb uptake in all treatments, whilst there were no significant effects on uptake of Ni across all treatments. All treatments were below the toxicity threshold levels for both Pb and Ni (Fig. 5c, d).

The highest uptake of Al (79.95 ± 21.2 mg Al kg^−^1) was observed for standard NPK, while the lowest (22.6 ± 3.7 mg Al kg^−1^) was observed for 20% Al-WTR (Fig. 5e). The Al uptake by maize observed for all treatments was below the toxicity threshold level of Al (200 mg kg^−1^). Uptake of Mn was highest (47.5 ± 4.6 mg Mn kg^−1^) in the 20% C + P and lowest (5.34 ± 0.32 mg Mn kg^−1^) in the 20% Al-WTR. The co-amendment, 10% Al-WTR + 10% C + P, resulted in higher Mn uptake compared with the unamended control (Fig. 5f).

Overall, the co-amendment 10% Al-WTR + 10% C + P resulted in lower uptake of Ni and Pb relative to sole compost treatments, whilst there were no significant differences in uptake of Al. Additionally, 10% Al-WTR + 10% C + P resulted in an increase in Zn and Cu uptake relative to all other treatments including the control (Fig. 5). 

## Discussion

### Characteristics of soil and amendment materials

The soil used in this study had high sand content (73%) and very low pH (4.0), which is considered very strongly acidic for Zimbabwean soils (Nyamangara & Mpofu, 1996). The high sand content means it has low nutrient retention capacity. The pH of 5.7 observed for Al-WTR is favourable for maize production, whilst that of compost, pH 4.8 is considered acidic and too low for maize growth. Soil pH has an impact on nutrient availability as it can render some essential plant nutrients unavailable for plant uptake whilst making others toxic for plant growth. Thus, the Al-WTR can play a critical role as a liming material, given that most of the soils in Zimbabwe as in many other countries in SSA are acidic. The Al-WTR’s relatively higher CEC than the control means that it has a relatively higher capacity to retain and supply plant nutrients compared with the soil. Total P of Al-WTR was higher than the soil and compost, but its available P was less than both, implying that most of the P in the Al-WTR was not readily available for plant uptake due perhaps to adsorption by the Al oxides in the WTR. Metal concentration of the Al-WTR was also higher than the control and compost but well below the European maximum permissible levels for heavy metals (Tóth et al., 2016). The Al-WTR is thus safe for land application as far as metal levels are concerned. The relatively high CEC in the compost proffers an advantage in nutrient retention capacity.

The similarity in post-harvest soil pH between the co-amendments (10% Al-WTR + 10% C + P and 10% Al-WTR + 10% C) and sole Al-WTR treatments is suggestive of the potential of WTR to modify soil pH (Hastings & Dawson, 2012). Al-WTR was able to mask the low pH due to compost in the co-amendment. The CEC of the residual soil due to the co-amendment was also higher than that for sole Al-WTR amended soils, and this was consistent with findings by Hsu & Hseu, 2011. This was attributed to the compost component in the co-amendment, which had a high CEC. From these results, it is evident that the benefits of combining Al-WTR and compost outweigh the benefits of sole use of these nutrient resources. The resultant lower concentrations of Zn, Pb, Al and Cd in both 10% Al-WTR + 10% C + P and 10% Al-WTR + 10% C in comparison with sole Al-WTR could be attributed to the presence of organic matter from the compost. Heavy metals become sorbed on the active sites on organic matter surfaces and form stable complexes with humic substances (Clemente & Bernal, 2006), making them less bioavailable. Even though metal levels for 10% Al-WTR + 10% C + P were elevated relative to the control and standard NPK, they were not bioavailable (Hovsepyan & Bonzongo, 2009). We attributed this to the favourable pH conditions proffered due to Al-WTR. Most metals including Al are bioavailable in acidic soils with a pH < 5.5. Al toxicity inhibits root growth. The significantly higher amounts of Ni, Al and Mn following the application of standard NPK mineral fertilizer could be linked to industrial processes during fertilizer manufacturing, which may have resulted in heavy metal contamination of the fertilizer. In the absence of organic matter, the metals become bioavailable. However, total metal levels in all the treatments were low in comparison with the European Community maximum limits. The high K in the control soil could be attributed to the granitic nature of the soil, which is inherently high in K (Nyamapfene, 1991).

### Impact of Al-WTR use in maize production

The observed decrease in plant growth and dry matter yield with the increase in concentration of Al-WTR suggests that Al-WTR amendment levels greater than 10% could be detrimental to plant growth. This is consistent with findings by Rengasamy et al. (1980) and Mahdy et al. (2007) where growth of maize in WTR amended soils increased until threshold application levels of 10 g/kg and 30 g/kg, respectively. However, compared to the control, the co-amendment of 10% Al-WTR, 10% C and P fertilizer resulted in higher maize growth and total biomass accumulation. This is in agreement with the work of Clarke et al. (2019), which also found higher wheat biomass yield due to combined use of compost and WTR as a soil amendment compared with unamended soil. Similarly, Hsu and Hseu (2011) reported that co-application of compost and Al-WTR resulted in higher dry matter accumulation of Bahia grass (*Paspalum notatum*), although in their case, the resultant yield was not significantly different to sole Al-WTR treatments. The enhanced growth and biomass noted could be attributed to the synergy in nutrient supply between compost and the Al-WTR. Although WTRs are typically low in P (Dassayanake et al. 2015), compost addition provided readily available P (due to its higher content of available P as shown in Table 1), whilst WTR provided N and a favourable pH for nutrient uptake. Land application of WTR for plant production is often constrained due to potential adsorption of P by the Al and Fe oxides normally present in WTR, making P unavailable for plant uptake (Babatunde et al., 2008; Bai et al., 2014; Norris & Titshall, 2012). The similarity in maize dry matter yield between 10% Al-WTR + 10% C + P and 10% C + P suggests that WTR can be used as a co-amendment with compost to increase maize yields and could thus reduce production costs by using half of expensive composts as the WTR is freely available.

The increase in maize growth and biomass accumulation due to the addition of P fertilizer accentuates the notion that the addition of inorganic P may, thus, help to alleviate problems of P fixation that leads to P deficiency in WTR amended soils (Basta, 2000). For example, Heil and Barbarick (1989) reported increased yield of *Sorghum bicolor* (Moench) in WTR amended soils through additions of inorganic P, whilst Lucas et al. (1994) showed that P deficiency in Fescue (*Festuca arundinaceae*) caused by the application of 40 g kg^−1^ alum sludge could be corrected by doubling the recommended P fertilization rate. In this study, a fixed P rate was used, which could have been too low to offset the negative P-fixing capacity of WTR. Further research may be needed to vary P rates and come up with optimal P application levels that can significantly offset the P-fixing capacity.

Poor plant growth and low biomass due to the unamended control attest that the soil used in the study is inherently infertile (Mapfumo & Giller, 2001; Nyamangara et al., 2000; Nyamapfene, 1991), with additions of fertilizer, and compost consequently improved maize plant growth and total biomass accumulation. The observed poor maize growth and biomass accumulation for standard NPK application, which is the common soil fertility management practice in Zimbabwe, could be an indicator of soil degradation. Degraded soils are known to show a general weak response to mineral fertilizer additions (Nezomba et al., 2015). Soil degradation due to poor soil fertility management is a major constraint to crop productivity in many smallholder farming areas in SSA (Mapfumo & Giller, 2001). Combining organic and inorganic nutrient resources has been proven to increase crop yields and nutrient efficiency in nutrient-poor soils (Mtambanengwe & Mapfumo, 2006; Vanlauwe et al., 2010) with other potential benefits to the soil physical, chemical and biological properties (Nezomba et al., 2015; Zingore et al., 2015). Research has also shown that farmers fail to access organic nutrients in sufficient quantity and quality to maintain the critical soil C levels for sustainable soil productivity (Mapfumo & Giller, 2001; Mtambanengwe & Mapfumo, 2006). WTRs can potentially contribute to soil C build-up in the long term because the organic carbon becomes tightly bound in the Fe and Al oxide matrix (Elliott & Dempsey, 1991; Novak & Watts, 2004). Hence, co-application of WTR with other organic nutrient resources could be a complementary option to increase soil organic matter to sustain crop production and at the same time protect the environment.

The low root-to-shoot ratios observed in the co-amendment compared to sole Al-WTR and the control signify better nutrient availability in the co-amendment. It is generally understood that when nutrients are available, plants allocate relatively less to the roots and more to the shoots and grain (Bonifas et al., 2005; Tilman, 1985) with exceptions where Mg, K or Mn are limiting (Ericsson, 1995). However, in P-deficient soils, higher root-to-shoot ratios are common. The highest root-to-shoot ratio due to the control is evident of the poor soil nutrient status. Root-to-shoot ratio could thus be used as an indicator of nutrient resource use efficiency in crop production.

### Influence of Al-WTR amendment on plant nutrient uptake

The inverse relation between soil and plant P due to the co-amendment of 10% Al-WTR + 10% C + P could suggest that some P could have been adsorbed and was thus unavailable for plant uptake. This could be attributed to the Al-WTR component of the co-amendment. Phosphorus deficiency in crops normally occurs due to slow release of labile P into the soil solution. Several studies have demonstrated that in WTR amended soils, readily available P can be converted to forms inaccessible by plant roots (e.g. Babatunde et al., 2008; Bai et al., 2014). Higher P uptake due to additions of inorganic P fertilizer was expected as the P in the fertilizer is readily available for plant uptake. Adding P fertilizers to soils amended with Al-WTR has a potential to reduce P sorption by the WTR, rendering the latter available for plant uptake. Babatunde and Zhao (2010) in their investigation on the kinetics of P sorption of alum WTR (Al-WTR) reported that initial sorption occurs on surface functional sites until these are saturated. This implies that added P fertilizer must satisfy these functional sites before it becomes available for plant uptake. However, this also implies additional P fertilizer cost on farmers. Cost–benefit analysis on long-term implications for WTR disposal into landfill *vis-a-vis* cost of P fertilizer will have to be done. Alternatively, P fertilizer subsidies can be made available to farmers willing to incorporate Al-WTR in their farms. The higher N uptake due to the co-amendment in comparison with Al-WTR treatments reinforces the mutual benefits in nutrient supply when Al-WTR and compost are used together (Clarke et al., 2019). The surge in N uptake observed in the co-amendment due to the addition of fertilizer P was likely a result of an increase in P availability and thus improved root development which enabled the plants to take up more N from the soil.

The high uptake of Ca, Mg and K accruing to 10% Al-WTR + 10% C + P relative to the control, sole WTR and compost treatments was also ascribed to the mutual relation in nutrient supply between the Al-WTR and compost, which had high levels of bases in addition to those from the WTR. The potential of WTR to supply cationic nutrients for plant growth and development has also been documented in the past (American Society of Civil Engineers et al. 1996; Dayton & Basta, 2001). More so, the high CEC of the WTR attests to its potential to hold and supply cations. The trend in uptake of the cationic bases also showed that maize has a higher demand for K compared with Ca and Mg. Potassium is required throughout the growth cycle as it plays a role in plant–water relations and regulation of ionic balances within cells. The superior response in uptake of Ca, Mg and K due to 10% Al-WTR + 10% C + P over standard NPK showed that a combination of Al-WTR + compost + P can be used as an alternative of the standard farming practice without any negative implications for uptake of Ca, Mg and K. Evidence has shown that a decline in the exchangeable basic cations leads to a decrease in maize yields (Mtangadura et al., 2017).

The relatively high uptake of Zn in the co-amendment was within optimal limits for maize production. Zn concentrations in maize plant tissue of between 20 and 60 ppm are considered sufficient (Tandon 1993). Deficiencies of Zn have been reported in African soils (Tagwira 1993; Manzeke et al., 2014; Kihara et al., 2020). Some studies have shown that integrated nutrient management including application of organic nutrient resources can increase plant Zn concentration (Manzeke et al., 2014; Yang et al., 2007); thus, WTR could potentially supply Zn in sandy soils (Dayton & Basta, 2001; Titshall & Hughes, 2005). The concentration of Cu in maize plant tissue due to the co-amendment was also well within the recommended limits of 300 ppm. From these results, Al-WTR can therefore supply safe levels of Cu. Although copper is required in minute quantities, it is important in plants for many enzymatic processes. The study also revealed that 10% Al-WTR + 10% C + P also enhanced Mn uptake by maize and that Pb, Ni and Al were all well below the threshold toxicity levels in maize plant tissue (Tandon 1993), signifying that compost and Al-WTR can be safely used as a soil amendment for maize growth without causing heavy metal toxicity. Based on these results, Al-WTR could complement other organic nutrient resources to supply micronutrients to the soil for plant uptake. The supply of micronutrients for plant uptake is important, given that micronutrient deficiencies are widespread in SSA arable soils (Kihara et al., 2020). This has great implications for human health—the high nutritional quality of edible plant organs improves human nutrition (Kihara et al., 2020; Yang et al., 2007). Improved human nutrition is important in Africa, given that over 200 million people are undernourished (FAO et al., 2018).

## Conclusion

The study demonstrated the superiority of combining Al-WTR and compost with P fertilizer in enhancing uptake of Zn, Cu and Mn by maize, which could provide an entry point for alleviating micronutrient deficiency in cereal-based diets in SSA. The study also showed that co-application of Al-WTR and compost together with the addition of inorganic P improved nutrient uptake, growth and dry matter yield of maize. The results also indicated reduced heavy metal (Pb, Ni, Al) uptake by the cereal crop in comparison with the unamended control, sole Al-WTR, sole compost treatments and standard NPK. There was also a decrease in post-harvest heavy metal content in soils co-amended with a combination of compost and Al-WTR relative to sole Al-WTR treatments. The significant increase in soil pH due to the co-amendment proved essential in decreasing bioavailability of heavy metals such as Pb and Ni and to reduce Al toxicity, which can be problematic in sandy soils. Overall, the study revealed that WTR can be co-applied with another organic nutrient resource such as compost for improved soil health (measured in terms of decreased bioavailability of potentially toxic elements Pb, Ni and Al) and increased crop production and environmental protection. We concluded that Al-WTR adds to the suite of available organic nutrient resources and can be co-applied with compost and mineral fertilizers to enhance soil quality and associated crop growth presenting a plausible alternative for re-using the product for soil improvement. Further research should investigate optimal inorganic P application rates to offset the negative effects of Al-WTR in P fixation as well as testing its agronomic benefits in field experiments.
